# Best practices for the collection and analysis of patient experience data from social media for patient-focused drug development

**DOI:** 10.3389/fmed.2025.1703923

**Published:** 2026-01-30

**Authors:** Philipp Cimiano, Nicole Brazda, Matthias Hartung, Cornelius Starke-Knäusel, Ana Lucia Schmidt, Maria Carmela De Vuono, Aditya Tyagi, Jürgen Gottowik, Raul Rodriguez-Esteban, Ben Collins, Krzysztof Wieckowski, Thierry Escudier

**Affiliations:** 1 Semalytix GmbH, Bielefeld, Germany; 2 Faculty of Technology, Bielefeld University, Bielefeld, Germany; 3 Boehringer Ingelheim GmbH, Ingelheim, Germany; 4 Roche Innovation Center Basel, Basel, Switzerland; 5 Chiesi Farmaceutici S.p.A., Parma, Italy; 6 Boehringer Ingelheim International GmbH, Ingelheim, Germany; 7 Pistoia Alliance, Wakefield, MA, United States

**Keywords:** real world evidence, patient experience data, social media listening, patient-focused drug development, guideline development

## Abstract

Patient experience data derived from social media captures the unsolicited conversations of patients and helps in understanding their subjective experiences with disease and treatments. By comparison, many other real-world datasets, such as electronic health records, have the drawback that they solely capture the perspective of health care practitioners. Regulators such as the FDA or EMA have recognized the potential of social media as a source of patient experience data that can inform patient-focused drug development. While social media has limitations, such as the reliance on patient or caregiver self-reporting, it allows us to understand the subjective perception and context of patients, how they experience their condition, its progression, existing treatments and how they manage these, which unmet needs they have, and how the disease affects their daily lives and activities. All this is crucial information that can inform drug development initiatives, and help substantiate relevant outcomes measured, both in clinical trials as well as in post-marketing evidence generation activities. This paper proposes best practices for Social Media Listening (SML) for the purpose of Real World Evidence generation along the following dimensions: purposes and objectives of a SML study, data collection, and data analysis. To illustrate how these best practices can be adopted, we showcase their application in a case study, aiming to unveil the key symptoms and comorbidities that diabetes type II patients face and how these affect their quality of life across an observation period of 24 months. We believe the proposed best practices will contribute to provide a rigorous methodological ground for the use of social media in generating patient experience data that can inform patient-focused drug development and could be accepted in regulatory processes.

## 1 Introduction

Social media is an important source of information patients use when seeking and exchanging health-related information about diseases or treatments. A survey conducted in 2020 showed that 55% of Europeans aged 16–74 have sought health-related information online, with a 21% increase since 2010 (1). According to an older study from 2013 conducted by the PEW Research Center, 59% of American adults use the Internet to look up health related information (2). A more recent study by PEW from 2019 on mobile phone use found that 6 out of 10 users access health related information via their mobile phones (3). For a large proportion of the public, young people in particular, social media sites are the first point of reference for obtaining health related information (4).

One of the main reasons patients use social media for health-related purposes is to satisfy emotional needs and supplement support they receive in interactions with their healthcare professionals (5). According to Smailhodzic et al. (6), the main uses of social media include obtaining different types of support: social, emotional, esteem, information, and network. One important effect of these uses is to increase empowerment in patients, in the sense of increasing the subjectively experienced level of control over their condition. Social media can also have a profound impact on patient-doctor relationships, increasing patients’ confidence and yielding a more equal communication between patient and healthcare professionals (HCPs) (7). Peers help the patient to have better understanding of HCPs and also empowers them to converse at eye level (8). For patients with rare, neglected, under-researched or stigmatized conditions, social media sites can be the only way to connect to peers (9).

Given the importance of social media as an empowering instrument, patients are willing to share very detailed and sometimes intimate information about their condition and treatment experiences. This is due to the fact that they can choose to remain fully or partially anonymous in their online interactions, and are thus willing to disclose more intimate details about their disease than they would be willing in a face to face conversation, e.g., with HCPs or researchers (10, 11).

Thus, social media has been identified as an important data source for generating patient insights that can inform patient-focused drug development (PFDD). PFDD, in general, aims to ensure that drug development reflects patients’ needs by integrating their lived experience and quality of life measures during disease and treatment [see also (12)] and to uncover unmet needs which could inform drug development to increase patients’ everyday living experience, as e.g., demonstrated in a meta-analysis study on an educational program against death anxiety for cancer patients by Su et al. (13). Many studies show that social media can help provide answers to important research questions related to patients’ needs, their symptoms, how symptoms affect their quality of life, etc. (14). The Food and Drug Administration (FDA) in the U.S., in their PFDD initiative,^1^ has highlighted that social media can be an important data source to generate patient experience data. More recently, a paper by the Big Data Steering Group of the Heads of Medicine Agencies (HMA) and European Medicine Agency (EMA) focused on the use of social media data in real-world settings, such as clinical care or the daily life of patients, to generate real-world evidence that could potentially support regulatory activities (15).

In contrast to other patient experience datasets, including Electronic Health Records (EHRs) as well as survey-based methods, the following characteristics make social media data unique as a source of real world evidence (RWE):

-    It provides the non-mediated and authentic experience of patients as voluntarily (and often anonymously) shared by them. This contrasts with EHRs or clinical notes, which are captured from the perspective of clinicians.
-    It enables the capture of longitudinal data for a historic cohort, allowing analysis of the evolution and journey of single patients in addition to recognizing (changing) trends at the population level.
-    It reflects the spontaneous conversations that patients have on topics that matter to them, overcoming biases inherent in other research methods that rely on pre-defined questions that might not reflect what is really important and matters to patients.

Social Media Listening (SML) refers to the “systematic use of manual and automated methods that extract relevant insights from social media data for observational studies” (16). While SML encompasses pharmacovigilance practices, this paper’s scope is focused on its application for patient experience research. Pharmacovigilance—the monitoring of social media for adverse event reporting—is a distinct, well established, highly regulated field. Moreover, SML signals could also contribute to automated drug-target interaction modeling and support computational pharmacology pipelines by incorporating the patients voice (17). This paper will concentrate on the methodologies for generating patient insights, rather than the specifics of adverse event handling.

Nevertheless, SML has specific limitations as discussed below (see chapter 2.3), such as the reliance on a patient’s self-reported conditions and the lack of independent medical verification as well as missing access to the clinical background of patients. SML is also affected by a number of biases including selection bias as only a specific subset of the population engages in online discussions, as well as reporting biases as information shared by patients online can be incomplete. These limitations are discussed below in detail but should be considered when SML is used for a specific research question.

Despite the mentioned limitations and (so far) lacking standard protocols for SML use, multiple SML studies have been carried out for various diseases including melanoma (18–21), metastatic breast cancer (22–25), COVID-19 or long COVID (26–29), and multiple sclerosis (30–33). Notably, SML has also been applied to rare diseases, such as complement 3 glomerulopathy (34, 35) or SLC6A1 disorder (36), which often pose challenges to established research methods due to relatively small patient populations. This variety indicates the suitability of SML as a highly flexible and versatile method to access patient experience data across a wide range of disease areas, irrespective of their prevalence.

Despite numerous studies demonstrating the effectiveness and cost-efficiency of SML, established guidelines and best practices are still missing (14). Such guidelines are essential to increase methodological rigor in the application of social media listening to inform PFDD and other regulatory activities. Sponsors, technology vendors and regulators need these practices to create capabilities for the execution and assessment of the results of such studies.

This article proposes a set of clear guidelines and best practices for algorithmically analyzing social media to extract insights about patient experience. Toward this objective, we review published papers to identify such practices, focusing primarily on re-analyzing publications from a recent review (14), where 63 articles were identified using SML for PFDD purposes and their research focus was identified. Benefits and drawbacks of the use of SML as a method to identify patients’ unmet needs were analyzed. Moreover, the perspectives of the three stakeholder groups, namely patients, life science companies and regulators, on the role of SML in drug development was assessed.

We focus on algorithmic methodologies that allow us to analyze social media data at scale, in contrast to manual analysis of small samples. We illustrate the application of best practices on an example study with the objective of understanding which symptoms and comorbidities impact the quality of life (QoL) of Type 2 Diabetes patients most, and how. Overall, our goal is to contribute to a constructive dialog between key stakeholders that are in the position to provide guidance on how SML studies should be conducted. In this sense, we follow the advice of the HMA/EMA Big Data Steering Group (15) that *“Early and continuous engagement with all stakeholders is essential for building skills and competencies, and laying a solid foundation for the use and adoption of social media data.”* Our contribution is to be understood as a first step towards laying these solid foundations.

BOX 1 **Key takeaways:**

- Social media plays an important role in patients’ lives, providing support at various levels; interaction with peers provides support that empowers patients to manage their disease better.
- Social media usage by patients for health-related purposes makes it a valuable source of unsolicited information.
- Social media listening (SML) refers to the systematic use of manual and automated methods that extract relevant insights from social media data for observational studies.
- Regulatory agencies such as the FDA and EMA have identified the potential of social media as a source of patient experience data to inform patient-focused drug development activities.
- SML has been successfully used to gather authentic patient experience data across multiple disease areas, including rare diseases.
- Best practices and methodological guidelines on how to execute SML studies are needed.

## 2 Applicability of social media listening for patient experience research

In the context of this paper, social media is defined in a very broad sense in line with Kaplan and Haenlein (37) as “*a group of Internet-based applications […] that allow the creation and exchange of User Generated Content*.” This comprises social networking sites such as X/Twitter, Facebook, Bluesky, but also support forums, discussion groups, online patient communities etc. We focus here on publicly accessible written content, and not on platforms that mainly rely on multimedia content, such as YouTube, Instagram, or TikTok.

SML is an approach that corresponds to an observational study setting. SML is characterized by the observation and analysis of the spontaneous and unmoderated conversations that patients and caregivers have online with their peers, without directly intervening or seeking to elicit them.

SML is mainly applicable in settings where it is crucial to get access and analyze the subjective experience of patients. Treatment experience, for example, is highly subjective, and preformulated questionnaires hardly cover all aspects that might lead to a decision for or against a specific treatment. Golder et al., for example, investigated the reasons for a statin treatment switch or discontinuation in the context of cardiovascular disease (38). The authors state that the exploration of such reasons is challenging and mostly undertaken in two ways: using cross-sectional surveys or in interviews. Surveys, although quick to perform, *“are at risk of recall and social desirability bias and limited by questionnaire design and delivery*,” whereas interviews are time consuming and *“are also prone to interviewer bias, relating to the way the interviewer asks questions and responds to answers, as well as their identity or behavior.”* The authors state that *“Social media posts tend to be contemporaneous to the event studied and are without the need for interrogation by a researcher, potentially reducing these biases.”*

Delestre-Levai et al. investigated the disease impact on quality of life of patients with Bronchiectasis (39) using SML, because, as they state, “*SML offers access to genuine opinions, emotions and comments: as the internet offers anonymity, patients are more willing to share their experiences, fears, concerns and challenges with others with the same condition.”* In another study, social anxiety disorder (SAD) was investigated using SML, which proved to *“provide a unique opportunity to understand the lived experience of individuals with SAD, for whom interacting with strangers is challenging”* (40).

SML studies, such as those mentioned, aim at a deeper understanding of individual patients’ needs, subjective feelings and insights into the patients’ everyday lives and mechanisms of decision making. Beyond this, the interaction between medical health caretakers and patients, as well as social or demographic aspects of disease or treatment experience are important research areas where SML can be applied. Depending on the research question in focus, patients’ self-reported information, as on their age, gender etc. was analyzed in previous SML studies (23, 27, 40–43).

There are, of course, research areas where SML for data collection is not applicable. These include topics where an objective ground truth independent of the subjective experience of patients is mandatory, e.g., tumor size changes after a certain treatment. Such questions are investigated in controlled clinical trials, where the results can be compared between arms of randomly assigned participants, allowing conclusions about causality. It is important to mention that many clinical trials also include the subjective patient experience by way of collecting Patient Reported Outcomes (PROs). SML, in contrast to asking pre-defined questions from PRO questionnaires, captures the spontaneous and unsolicited experience of patients.

As discussed in chapter 2.3, SML has limitations which must be considered before choosing this observational research method (e.g., selection bias, reporting bias etc.).

### 2.1 Research settings

The advantages and disadvantages of SML in comparison to other research methods, interviews and surveys in particular, are summarized in Table 1, taking into account the dimensions proposed in earlier work (44, 45): (i) geographical coverage, (ii) time and cost, (iii) burden on the subject, (iv) analyzed time ranges, (v) extraction of answers, and (vi) probability of identifying new concepts. This table was originally derived from Humphrey et al. (44) with permission, and adapted for the purpose of this paper. The authors conducted a comparing study on the three different methods to collect patient experience data to generate a conceptual understanding of a disease. Details of the methodology and qualification can be found therein.

**TABLE 1:** Suitability and limitations of SML, interviews and questionnaires in order to gather patient experience data [originally derived from Humphrey et al. (44) and adapted for this article].

Topic	SML	Interviews	Questionnaires
Geographical constraints	Low	High	Setup dependent
Time and cost	Automated or mixed methods for data collection and analysis are not time consuming regarding the large amount of obtained data	Very time consuming, high costs for 1:1 interviews	Reading and analyzing questionnaires is very time consuming
Availability of clinical background/data	Not applicable due to anonymity	High, clinical data might be collected as well	Depending on concept, medical records might also be collected
Level of burden to subject	Low, patients report voluntarily	High stress and pressure might be felt during a 1:1 interview	Low if done anonymously
New concept identification	Very high, due to open nature of data collection, completely new insights can be generated	Low chance to find new concepts since questions are normally standardized and predefined	Very low new concept generation due to rigid structure
Representability in whole patient population	Self-reporting biases may affect representability, but quantitative assessments can be applied	Representability is strongly dependent on patient selection. Small data set decreases representability	Representability can be increased by patient selection and data volume adjustment
Access to perspectives from patient-related individuals	At low cost but based on their level of online engagement	Needs to be predefined in the study and increases costs considerably	Increases study complexity
Data time range	Retrospective data analysis allows analysis over multiple years. However, the sequence of patient-authored narratives does not necessarily follow the order of events in real time. Patient-generated content over a longer timeframe might contain inconsistencies and contradictions due to changing perception along the patient journey (better episodes vs. worse episodes)	Interviews reflect a momentary situation, and past experiences can be altered by biases inherent in memory recall. Sequence of events can be clarified in the interview but relies on ability of participants to recall accurately	Depending on the setup, e.g., repetitive questionnaires with the same patient population, time range can be large. Sequence of events can be requested in the questionnaire but depends on participants’ ability to recall past events
Difficulty in extracting information from the data	Technology of data extraction must be chosen depending on the research question	Difficulty in data extraction might result in subjective perception of questions and answers of interview partners	Data extraction and analysis is part of the questionnaire design, therefore not difficult
Biases at play	Selection bias (social media presence), negativity bias, naive audience bias	Selection bias, reporting bias, recall bias, framing bias	Selection bias (patient population), question selection
Complexity for patients	Low since use of social media is simple, words used by patients are their own words. Do not require to be tech savvy	Low but requires explanation and attention from the interviewer	Medium, requires clear instructions if patient is alone to answer

With respect to geographical coverage, interviews and surveys are limited to subjects recruited from pre-defined locations. SML, in contrast, can be applied to collect and analyze data globally. Segmentation per country/location, however, requires the availability of corresponding geographic metadata, which might not always be available.

Regarding time and cost, data collection and (automated) analysis can be performed at low cost compared to the effort of performing surveys or 1:1 interviews, because SML does not generate new data, but repurposes existing data, which consumes substantially less time, once the according algorithms are established and tested. Notably, if social media data analysis was performed manually, SML would presumably become highly labor-intensive, which is why automated analysis is suggested in these best practices.

Considering the burden on the subject, traveling to a hospital to participate at an interview (46) or the completion of surveys can represent a significant burden for patients and data subjects (47). SML, instead, is a passive observation method that analyzes information patients and caregivers have already voluntarily provided on social media sites.

With respect to analyzed time ranges, interviews and surveys typically capture only a snapshot of the patient experience, usually past events and experiences, but answers are limited by patients’ ability to recall. SML has access to historical data and can collect and analyze data from different time ranges as needed. Nevertheless, it must be considered that, due to their condition, patients might be unable or unwilling to share their experiences on social media. Further, patients might share information also very selectively online depending on what is relevant to them at the moment of posting.

Regarding the extraction of answers, this can be straightforward for multiple-choice survey questions with predefined answer options. Data from open-ended survey questions and 1:1 interviews might require an additional subjective interpretation step. SML typically requires selection of a suitable algorithmic solution to extract answers/concepts from unstructured data or needs to rely on manual coding according to a certain proprietary coding scheme specific for the research objective.

The probability of identifying new or previously disregarded concepts of interest (COI)^2^ is very high in SML studies since the patients’ voice is processed directly with minimal constraints. New concept discovery is less likely in surveys where questions are pre-defined and answering options might be even determined up-front. Interviews offer the chance to find new concepts or create deeper understanding of the patients’ experience, but depend on the subjective skills, experience, and training of interview partners. On the other hand, the subjective skills of researchers selecting and optimizing algorithms for SML can also be a limiting factor in SML. Semi-structured interviews offer the possibility to ask for further clarification if a patients’ report seems incomplete. SML does not offer this opportunity, since all reports are already completed when data is collected, but due to the large volume of obtained data, e.g., by thousands of patients, the probability to find patients’ posts containing the desired textual depth are high.

Fortunately, SML can, at any point of the study, capture the perspective of individuals who are related in different forms to the patient, such as caretakers, friends, partners, and relatives, and which may not be in scope in other studies, unless they were explicitly designed to include this group.

Important limitations of SML include the fact that there is no controlled recruitment process and inclusion relies on patients’ self-report. SML is limited to patients who have an online presence in social media.

Further, SML lacks access to the complete clinical history and background of subjects. Given that consent is typically not obtained and patient privacy needs to be protected (see more detailed discussion in section 3), subjects cannot be linked to other existing clinical histories. This is different to interview and survey settings, where subjects are typically actively recruited and can provide consent to access their medical history.

All these methods for RWE generation are affected by different biases, mainly selection bias, but also by reporting bias concerning information that patients choose to disclose. SML might be affected by negativity biases because patients might focus on negative experiences. However, surveys and interviews are also affected by selection biases, concerning the sample of patients recruited. Results from interviews and surveys are affected by recall bias as patients might not accurately remember past events and might further be biased by question selection and framing.

SML offers the advantage of accessing very large data sets, allowing analysis of a wide time range of patient experience. Interviews are limited to a small number of patients and reflect a present snapshot. In this regard, questionnaires represent a compromise between these two methods, since data volume and time range of data collection can be predefined.

Finally, there are limitations in terms of performing quantitative analysis in the context of SML as data can be very incomplete. These limitations are discussed in more detail below in section 2.3.

### 2.2 Research questions suited for social media listening

**FIGURE 1:**
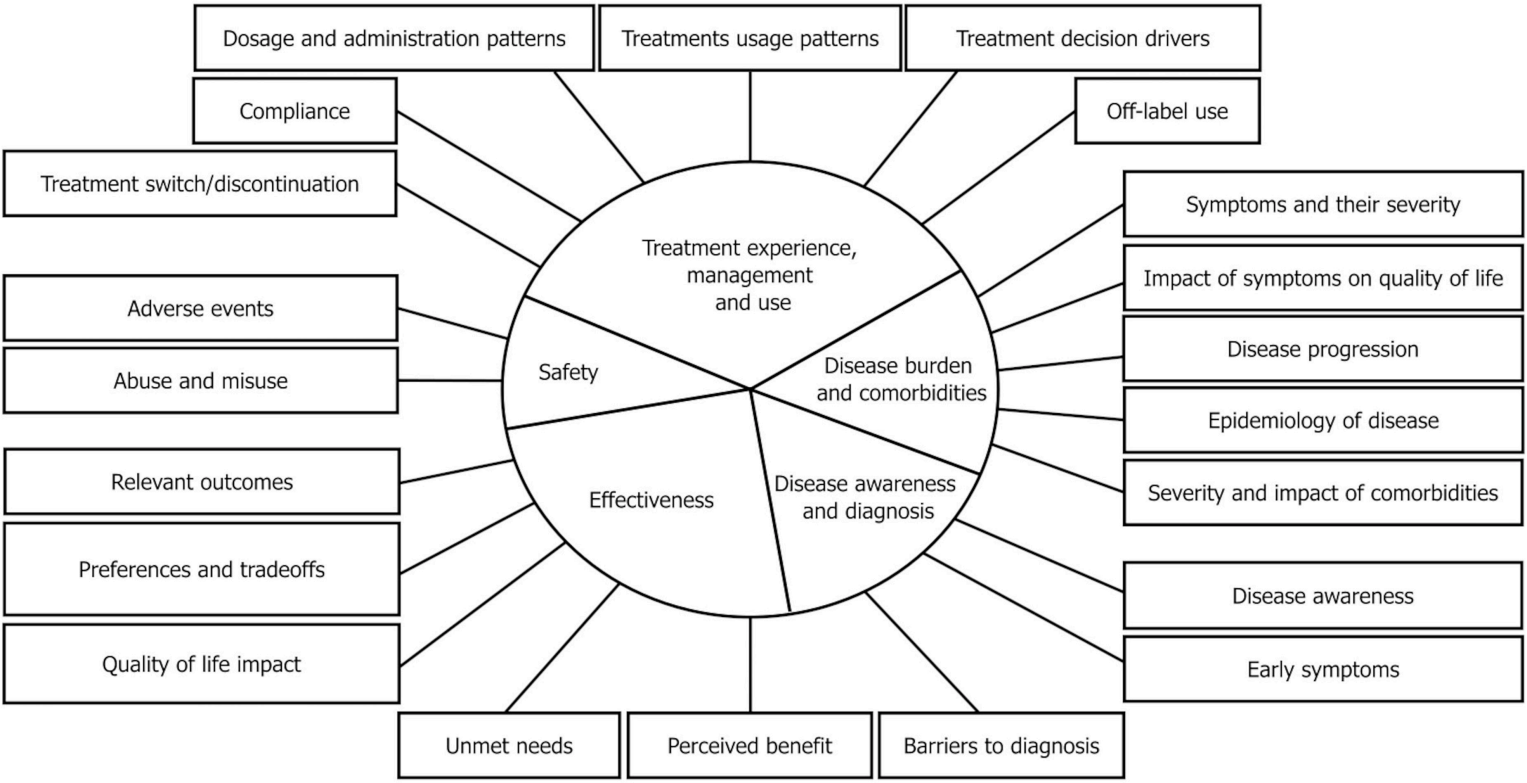
Schematic representation of main categories of research questions addressed in 63 scientific publications using SML for patient experience data analysis. The research topics were clustered under five top categories for descriptive purposes. This schematic representation is not intended to be exhaustive and may be supplemented as needed.

We determine the most prototypical areas for research questions by re-analyzing 63 publications discussed in a previous study (14) complemented by very recent literature. We find that most research questions addressed by SML approaches fall into the categories represented in Figure 1: (i) Disease burden and comorbidities, (ii) Treatment experience, management and use, (iii) Treatment effectiveness, (iv) Safety, and (v) Disease awareness and diagnosis.

In order to define the research categories, we have relied on the types of patient experience that the FDA has defined in their first guideline on patient-focused drug development [“Guideline 1: Collecting Representative and Comprehensive Input (48)]. The suggested types of patient experiences include (page 4, Section B): (i) signs and symptoms, (ii) effects of the disease on patients’ quality of life as well as disease progression, (iii) experience with treatments, (iv) subjective perception of outcomes, (v) impact of disease, treatment and outcomes, trade-offs between benefit and risks.

We reviewed 63 SML studies considering the above categories. In an iterative process, research question categories were adapted and agreed upon within a group of experts from the Pistoia Alliance Community of Experts (CoE) to harness real world data from social media for patient-focused drug development. The defined categories are not fixed, rather they can be adjusted and serve as a guidance. Some research questions, as e.g., “Understanding key topics, impacts on QoL, and unmet needs in those living with dry eye disease as reported by patients.” might fit in multiple categories, e.g., in this case, either “Effectiveness” and also “Disease Burden.” These categories serve as a descriptive tool for clustering questions rather than definitions.

*Disease burden and comorbidities* describes research topics aiming at a deeper understanding of symptoms, comorbidities, progression, prevalence, and the impact of the disease on the patient’s daily life. For instance, several authors have used SML to understand the perceived severity of symptoms (42) while others have investigated the impact of symptoms on daily living and QoL of patients (18, 39, 40, 42, 43). Beyond this, studies have also investigated the impact of disease on the environment of patients and their caregivers (49).

*Treatment experience, management and use* are commonly discussed topics on social media. Thus, SML can help to identify patterns of use and help to answer questions around schedule, frequency and dosage of treatments. It can also help to understand what factors negatively affect compliance and adherence. For example, several studies have focused on understanding reasons for treatment switching and/or discontinuation (38, 50, 51). SML has further been used to understand the decision-making process of patients and factors for selecting a treatment (52).

*Effectiveness* refers to the subjectively perceived improvements that patients experience due to treatments, SML studies can help to understand treatment gaps patients experience as well as the subjectively perceived benefits beyond purely clinical outcomes/endpoints. For instance, some researchers have used SML to uncover unmet needs of patients (42) or the importance of specific symptomatic or functional outcomes (53). Importantly, patients’ preferences and trade-offs they are willing to make and the risks they are willing to take are investigated (54). Overall, SML studies allow us to understand how patients define the effectiveness of treatments and which outcomes they consider valuable. This can inform clinical study endpoint selection or post-marketing RWE studies.

*Safety* is a key area of investigation that includes research on adverse events (19), off-label use, and instances of misuse or abuse of treatments (55). Because social media platforms allow for anonymous sharing, patients may be more open about sensitive or stigmatized experiences, thus reducing reporting bias and highlighting safety concerns that might otherwise go unreported.

*Disease awareness and diagnosis* comprises research on how patients recognize symptoms, seek medical attention, and navigate the diagnostic process. Studies in this area aim to uncover which symptoms prompt individuals to consult healthcare professionals, what barriers exist to receiving a timely diagnosis (56), and how patients’ awareness of a condition evolves over time (28). Furthermore, SML can be a valuable tool for identifying gaps in public knowledge and opportunities to enhance early detection through improved disease awareness.

In Table 2, we provide examples of studies for each of the above-mentioned categories of research questions.

**TABLE 2:** Common research questions in publications using SML for patient experience data.

Research question category	Research question topic	Research objective	References
Disease burden and comorbidities	Impact of symptoms on quality of life (QoL)	Understanding the most frequently discussed topics along the patient journey and impacts of melanoma.	(18)
		Comprehending patients’ perspectives and opinions on symptoms, treatments and (emotional) burden of Bronchiectasis.	(39)
		Understanding the experience of adolescent SJIA patients and those of their parents based on their own social media posts.	(49)
	Symptoms and their severity	Collecting the most frequent and most severe symptoms of COPD.	(42)
		Comparing the interpretation of the burden of atopic dermatitis among patients and physicians.	(95)
	Epidemiology of disease	Assessing the social determinants of health factors of marginalized racial/ethnic US population groups that were disproportionately impacted by the COVID-19 pandemic.	(96)
	Impact of symptoms on daily living	Exploring what lung cancer patients, caregivers, and HCPs are discussing on social media platforms regarding burden of illness, epidemiology, patient characteristics, treatment patterns and compliance, QoL, predictors of outcomes, and effectiveness/safety.	(43)
		Understanding the impacts on functioning or daily living for individuals with social anxiety disorder (SAD).	(40)
	Disease progression	Analyzing challenges related to the progression, worsening, or recurrence of advanced bladder cancer.	(97)
Treatment experience, management and use	Treatment decision drivers	Understanding AML or MDS patients’ and caregivers’ experience regarding their disease and treatment, if they are not eligible for intensive chemotherapy.	(52)
		Comprehending perspectives that potentially influence the treatment decision among patients with psoriasis.	(98)
		Identifying the most influential users in the healthy diet discourse on Twitter/X and exploring the characteristics of these users in order to inform public health communication strategy and interventions.	(99)
		Exploring patient knowledge, attitudes, and beliefs regarding biologic therapies in ankylosing spondylitis (AS).	(100)
	Treatment switch/discontinuation	Assessing the reasons for discontinuation or switching of statin therapy as reported by patients on social media.	(38)
		Identifying instances of drug switching in social media posts, as well as a method for extracting the reasons behind these switches.	(51)
		Understanding patterns of treatment switching in multiple sclerosis (MS).	(50)
	Compliance	Understanding non-compliance, switching between treatment options or technologies, or discontinuation of treatment options in adult and pediatric amblyopia patients.	(101)
	Treatments usage patterns	Collecting topics of relevance before and during Invisalign treatment (indication: crooked and crowded teeth), and pertaining sentiment.	(102)
Effectiveness	Unmet needs	Understanding key topics, impacts on QoL, and unmet needs in those living with dry eye disease as reported by patients.	(103)
	Relevant outcomes	Detecting the different topics discussed by patients and relating them to functional and symptomatic dimensions assessed in the internationally standardized self-administered questionnaires used in cancer clinical trials.	(53)
	Preferences and tradeoffs	Understanding patient perspectives on medical product risks and living with different medical conditions.	(54)
	QoL impact	Studying the patient journey in a type of liver disease by exploring the perspectives of patients, caregivers, friends and family, and healthcare professionals, including QoL impact and unmet needs.	(104)
Safety	Perceived benefit	Assessing MS patients’ real-life experiences with medicinal products during pregnancy as well as their struggle in comprehending the benefits and risks associated with these products.	(33)
	Adverse events	Uncovering the most common concerns among patients with adjuvant and metastatic melanoma receiving immunotherapy or targeted therapies and their caregivers, including treatment preferences regarding reduced risk of adverse events.	(19)
	Abuse and misuse	Analyzing potential misuse or nonmedical use of bupropion and two comparators: amitriptyline and venlafaxine.	(55)
Disease awareness and diagnosis	Disease awareness	Understanding the negative consequences that arise when there is a disconnect between official health communications and research, and that of the actual lived realities of those suffering from Long Covid.	(28)
	Barriers to diagnosis	Collecting barriers to diagnosis of rare eosinophil-driven diseases, including mentions of HCPs overlooking symptoms that ultimately supported clinical diagnosis.	(56)

### 2.3 Limitations

While we have highlighted that all research methods discussed have advantages and disadvantages and suffer from biases, in this section we discuss limitations that are specific for SML as an observational research method. For one, SML fundamentally relies on patients’ or caregivers’ self-report, and access to clinical/medical information is usually not provided. The accuracy of social media reports can be compromised due to lack of (medical) knowledge and use of colloquial/non-technical vocabulary. Patients describe symptoms “as they experience them” in their own words, and not necessarily in clinical/medical terms. While this holds true in interviews as well, 1:1 interviews allow for direct clarification in case of ambiguities. It might also be possible that patients share very personal experiences in private fora or communities and, therefore, SML in public communities could miss important aspects.

Nevertheless, it has been argued that this “lay perspective” of patients’ report in social media allows an unique view on the disease/treatments from the point of view of those affected, providing access to the patients’ language, disease conceptualization, weighing of outcomes, etc. (57).

Obviously, SML is limited to topics discussed by patients, and, therefore, incompleteness of data is a fundamental methodological limitation. Patients might experience a symptom, although they do not report it, which might imply its low relevance or burden. Therefore, studies need to be very explicit about what they infer from missing data and how they handle reporting bias, which are both known problems. Statistical methods, where reporting habit, frequency and detail depth of patients’ reports are considered test variables can be applied to test defined hypotheses, but need to be planned thoroughly. The use of mixed methods should be considered.

Some research questions requiring very detailed information, e.g., on the exact timing of symptoms, precise dosage and administration frequency of drugs, the precise treatment schedule, the exact tests by which a diagnosis was made etc. might not be answerable using SML as it is uncommon to find this level of accuracy and explicitness in patients’ comments, unless it is a focus of the online discussion. Moreover, SML is not suited to provide mechanistic insight into biological processes like preclinical studies, e.g., (58), or directly compare treatments at a level of sophisticated controlled clinical trials, e.g., (59), but complement it.

As other RWE generation methods, SML is affected by population/selection bias. Patients who do not report or share their posts publicly on social media, might have different opinions, introducing potential bias. Patients might outsource information that might compromise their privacy or security if that content is made public for anyone to see. As some patients might be active on multiple platforms, there is a risk that some author profiles might be duplicated when data is collected in different platforms. More recently, the massive adoption and proliferation of generative AI has increased the chance that some patient profiles are fake. There are, unfortunately, no off-the-shelf solutions that can be applied to eliminate the risk of fake post consideration. However, AI-detection tools are evolving and could be applied for data cleaning yet still need to mature.

Providing solutions for these limitations is clearly out of scope for this article, yet it is important to highlight them when discussing the benefits of SML in comparison to other methods. Studies using SML need to position themselves clearly in how far they deal with these limitations.

### 2.4 T2DM example case study: choosing research questions and settings

To illustrate the points raised, we describe a case study with the objective to understand the subjectively perceived impact of symptoms and comorbidities on type 2 diabetes mellitus (T2DM) patients. Common T2DM comorbidities or complications include hypertension, lipid disorders, cardiovascular-related conditions (e.g., coronary heart disease), microvascular conditions, and depression (60). The research objectives of this study include:

1. What conditions (symptoms, comorbidities, complications, etc.) do patients experience and how severe are they?
2. How do these conditions affect patients’ daily life?

Notably, the questions do not solely address quantitative aspects, such as number of patients with a certain morbidity, but also the degree to which patients perceive the comorbidity as burdensome. The second question is more open-ended regarding “how” patients are affected by the comorbidities, representing an explorative question that requires identifying key concepts with minimal preconceptions. As the research questions revolve around understanding the subjectively perceived patient burden, they fit well into the types of research questions and settings that can be addressed with an SML study, belonging to the topic of disease burden (see also Table 2).

**Key takeaways:**

- SML methodologies correspond to observational study design settings.
- SML is ideal to support explorative research and find new or previously disregarded concepts from subjective patient perceptions.
- SML is a cost-effective method that allows to collect data longitudinally and globally, it offers high immediacy since real-time posts can be included.
- SML allows insights into rare diseases and rare events which cannot be detected in small data groups or segmentation of patient groups.
- Suitable research questions for SML approaches include capturing key concepts of treatment management and experience, disease burden including comorbidities, disease awareness and diagnosis, perceived effectiveness, and unmet needs.
- Access to the naive and lay perspective of patients is a strength of SML as it allows to see the disease from the eye of patients. As a drawback, statements might be inaccurate and difficult to interpret from a medical perspective.
- Key limitations of observational study designs are self-reporting bias, incompleteness of data, and lack of causality.
- It is important to make explicit assumptions under which one interprets the absence of mentions of certain symptoms or events.
- Questions not answerable with SML may include questions related to exact timing of events/symptoms, dosages, treatment schedules, causal effects, etc.

## 3 Collection of patient experience data from social media

When collecting data from social media, a recruitment-based approach is generally not possible. This means that, typically, there is no possibility to identify *a priori* all the patients that satisfy the inclusion criteria without processing the data first. This implies that a sufficiently large sample of (potentially relevant) data needs to be collected and filtered down to the population of interest.

As the first step to identify the best strategies to develop these guidelines, published strategies for data collection were analyzed, as reviewed in Cimiano et al. (14). Then, a panel of experts with substantial experience in developing and applying SML to answer research questions was convened within the Pistoia Alliance CoE.^3^ The authors of this article are members of this community. Each step for data collection and analysis was discussed within the panel and agreed upon to present the following best practice guidelines.

The relevant steps for data collection are the following:

1. *Data source identification:* To maximize the likelihood of finding relevant data that match the population characteristics, several relevant sites, fora and support groups need to be identified. If the resource is public, a good strategy is to read a sample of posts to determine the relevance of the data source. Selecting several data sources represents a sampling strategy from a methodological perspective, as we select some sites and fora out of many that exist. Sources can vary in terms of specificity for the analysis at hand and the lack of sufficiently specific sources may lead to the selection of more general sources (e.g., Reddit, Twitter/X). Potential biases incurred by source selection should be made explicit. Patient forums that require registration and are moderated will feature a higher level of quality and less noise (e.g., in terms of fake accounts) compared to using a completely open social media application such as X/Twitter or TikTok.
2. *Data extraction:* Regarding data extraction, there are different alternatives that depend on the data provider and the interfaces to access the data. One possibility is to extract the complete content of the identified fora to focus the analysis on the complete context and history of posts for each user and not on single posts that contain relevant keywords. This will allow the aggregation of all the posts of a single patient to get a complete picture or even perform longitudinal analysis that is not possible when collecting only single posts. However, collecting the complete dataset might not be an option for some data providers/communities/networks, either because of data volume (e.g., Twitter/X) or technical limitations or restrictions from the data provider. In these cases, we might be restricted to access the data matching a particular query or set of keywords. This method will bias the data as the filter imposed will reduce the recall.
3. *Data storage:* After extraction, data needs to be physically stored for further processing and analysis. At this step, the data might not yet be pseudonymized, so technical and organizational measures (TOM) need to be put in place to ensure that only a minimum number of administrators have access to the data before pseudonymization. Once pseudonymized (see step 4 below), data is transferred into a dedicated database or storage system on which processing will be performed, the non-pseudonymized data should be deleted.
4. *Data pseudonymization:* To protect the identity and integrity of data subjects it is recommended to pseudonymize data by removing any personal attributes including user IDs, user addresses, names, telephone numbers, email addresses, etc. and introducing a new identifier that has no relation to any personal attributes of the user. A randomly generated identifier or hash value are viable options here. There are several vendors on the market offering pseudonymization solutions off-the-shelf.
5. *Algorithmic coding:* Different solutions and approaches can be used to automatically code the data with key concepts, particularly to filter the extracted data. Most approaches involve natural language processing (NLP) or artificial intelligence (AI) systems that have been trained to recognize key concepts (see more details on this in section 4). However, approaches that apply manually defined patterns to the data are possible. Some concepts that could be detected to allow for data filtering are the following: demographic information about patients (age, gender, geography), diagnosis/condition, symptoms/comorbidities, treatments, and phenotypic characteristics.

In general, data can be collected retrospectively or prospectively. While retrospective collection refers to data from conversations in the past, prospective collection happens in a “live” mode as the content is generated and potentially relevant events, such as the release of a new treatment, unfold.

In collecting data, it is mandatory to adhere to applicable data protection laws. While it is not feasible in this article to review all regulations worldwide, we discuss the most common considerations in reference to the General Data Protection Regulation (GDPR),^4^ which protects EU citizens all around the globe. The essential concepts discussed also apply to other data protection frameworks (e.g., CCPA^5^ in the US/California, PIPL^6^ in China, 2020 Privacy Act^7^ in New Zealand, etc.). The GDPR distinguishes two important roles, the role of the *data subject* and of the *data processor or controller*. The data subjects identity must be protected and the data subject has several rights including objecting to data processing, revoking consent, receiving information on processing, deleting data, etc. The data processor or controller is responsible for design decisions and implementation steps involved in the data processing pipeline. The basis of the GDPR is that there must be a legitimate purpose for processing the data that needs to be balanced with respect to the rights of data subjects to protect their identity, privacy and integrity. According to Beauchamp and Childress’s principles of research ethics, researchers have an obligation to ensure no harm comes to participants, and that the research will have potential benefits for the target group (61). The importance of the research question from a societal perspective and whether other methods would be applicable to answer it should be considered and balanced with respect to the risk involved for the human subject concerning norms of autonomy, integrity and equality (14, 62).

The objective to research relevant aspects of the patient experience is a legitimate purpose that needs to be balanced against the rights of the data subject. Assumptions of the legitimacy of data usage and processing regarding its sensitivity for the subject should be made explicit in a Data Protection Impact Assessment (DPIA) or similar document, although it has been argued that sensitivity level could be regarded as low if data has been *“manifestly made public”* without any protection [see e.g., GDPR Art 9. 2 (e)]. A recent review on ethical considerations in the context of automated text mining of social media data has suggested that SML for health research can be ethical even without informed consent since social media data might be regarded as “public data” (63). The Economic and Social Research Council (ESRC) in the UK considers ethical the foregoing of patient consent as long as a study is not *“undertaken lightly or routinely”* (64) and the objectives addressed are of “*social significance which cannot be uncovered in other ways”* [ESRC, Framework for research ethics, 2010 (64)]. In addition, the Ethical Guidelines of the Association of Internet Researchers present strategies to address the issue of lack of informed consent in Big Data projects in The Internet Research Ethics 3.0 guidelines,^8^ where the authors emphasize that consent is impracticable in Big Data projects and pseudonymization should be used for individual subject protection. Pseudo-anonymizing content and putting technical and organizational measures (TOM) in place so that data subjects cannot be re-identified is current best practice in SML. Any personal attributes as user ID, names, addresses etc. should be removed before analysis. Moreover, researchers should be instructed not to search for content on the Web as this might re-identify the specific persons.

In the case of SML, obtaining consent for social media data use would require contacting each patient in the dataset, although the patient might be excluded in the final analysis (see above). Patients would need to be contacted manually via the respective platform/site, which would require the creation of an account (often forbidden for non-patients), if direct messaging was possible at all. Therefore, consent is usually not obtainable.

Another consideration regarding contacting patients for consent might be, that the act of contacting users may bias their own future online actions as well as those of their peers, as they may start behaving differently, changing the very nature of online conversations in a way that could be detrimental to future SML activities by any researchers.

An important question is certainly whether patients whose online posts are used within a SML study are human (study) subjects whose identity needs to be protected or content authors that need to be credited for their content [see discussion by Snee (65)]. A relevant question in this context might be whether patients’ posts have actually to be treated anonymously or whether authors cannot be directly credited as authors of the content (65, 66).

Regarding the question of whether ethics approval is needed to carry out a SML study, views are more heterogeneous. It has been argued that no ethics approval is needed when the method is purely passive and there is no direct interaction with social media users, so that they cannot be regarded as human subjects (63). However, ethical approval was considered necessary for research using data from closed groups, engaging in direct contact with users, when conducting any kind of intervention through social media, if research was specifically about users who are under 18 or lack capacity, if users could be identified from the study publication or dataset, if multiple sources of data are being linked, or if, following consultation, it is assessed that there are reasonable risks of potential harms or stigmatization occurring. Likewise, researchers should gain consent from social media users under those circumstances. This is in line with regulations, such as that concerning institutional review boards (IRBs) in the US, which do not require ethics approval for observational studies of public information. Above all, the narrative review clearly highlights that the terms and conditions of every data source need to be abided by.

### 3.1 T2DM example case study: applying best practices for data collection

To collect data from a T2DM population we focused on public support groups, forums and networks in English language. These were obtained through a search including a number of data providers. We relied on available search interfaces to query for online communities containing the following keywords: diabetes, DT2, DT1, type 1, type 2, HbA1c. A manual analysis of a sample of posts for each forum was performed to ensure relevance of the data.

As a result, we included a list of 32 online forums in our research case study (Supplementary Table 1). The sources comprise larger online forums such as *reddit.com* as well as smaller targeted communities such as *diabetes.org.uk*.

Once a site was determined as relevant, the complete site was crawled via the APIs of the data providers, selecting only sites whose terms and conditions do not exclude crawling for non-commercial purposes. The downloaded data was stored in a database to which only system administrators have access to. Data was automatically pseudonymized using state-of-the-art anonymization services, removing usernames, names and any other personal identifiers including emails, telephone numbers, addresses, etc. After pseudonymization, the data was transmitted to a second database for analysis. The organizations involved in this project have DPIA or similar documents in place under which they can carry out analysis of public data provided that there is no harm on the data subject. Given that the content was publicly shared without any password protection, the further analysis of this public content is possible without causing harm to the authors. As we did only process public and pseudonymized data and did not specifically target vulnerable groups, minors etc. it was regarded as acceptable to perform the analysis despite having explicit consent. We ensured that the terms and conditions of these fora allow for crawling and mining for non-commercial purposes or ensure to enter licensing agreements with a data provider.

**Best practices/recommendations:**

- Identification of relevant sites/forums/groups can be considered a sampling step that needs to be carefully carried out to manage biases.
- Prefer selecting different social media sources to mitigate source bias and try to prioritize collection of data from public forums.
- Collect whole forums instead of single isolated posts to reduce bias by search terms and obtain the full context/history of patients.
- Follow and adhere to source terms and conditions.
- Follow data protection regulations and principles, create a Data Protection Impact Assessment or similar document that details how harm to social media users is minimized.
- Put technical and organizational measures (TOMs) in place to protect the integrity and privacy of data subjects.
- Process data to ensure pseudonymization, proper data storage and key feature extraction (demographics, diagnosis, comorbidity, treatment).
- Put safeguards to ensure that analyzers of the data do not search for quotes from the data online, as this might lead to re-identification of individuals.
- Put measures in place to detect and remove fake accounts/users/advertisements from the data.
- Ensure that the research objective is of public interest or otherwise obtain consent for a study when using closed group data.
- Obtain ethical approval in any of the following cases: (i) using data from closed groups, (ii) engaging in direct contact with users, (iii) when conducting any kind of intervention through social media, (iv) if research was specifically about users who are under 18 or lack capacity, (v) if users could be identified from the study publication or dataset, (vi) if multiple sources of data are being linked, or (vii) if, following consultation, it is assessed that there are reasonable risks of potential harms or stigmatization occurring.
- Only process data of groups that can be assumed to be aware of the sensitivity of information they are sharing publicly on social media.

## 4 Algorithmic analysis of patient experience data from social media

In the context of SML, data analysis relies on algorithmic coding of key variables in patient-authored text based on different natural language processing (NLP) approaches. While manual coding is feasible for smaller samples of data, our focus here is on scalable data analysis methods that allow us to tap into large data collections to capture a population in a representative way.

Algorithmic analysis of patient experience data from social media aims at cohort-level analysis that allows us to treat social media data similarly to data from other observational studies where we have a cohort of patients, and we can record observations that are attributable to single uniquely identifiable (albeit pseudonymized) individuals. This cohort-level analysis is different from a “signal-level” or “trend-level” analysis where mentions of entities (brands, symptoms, treatments etc.) are quantified without attribution to a specific member of the cohort. Signal-level analysis is, therefore, regarded as more shallow than cohort-level analysis, which might be the better choice for PFDD when it is important to link concepts to identifiable members of a cohort.

### 4.1 Conceptual patient experience model

**FIGURE 2:**
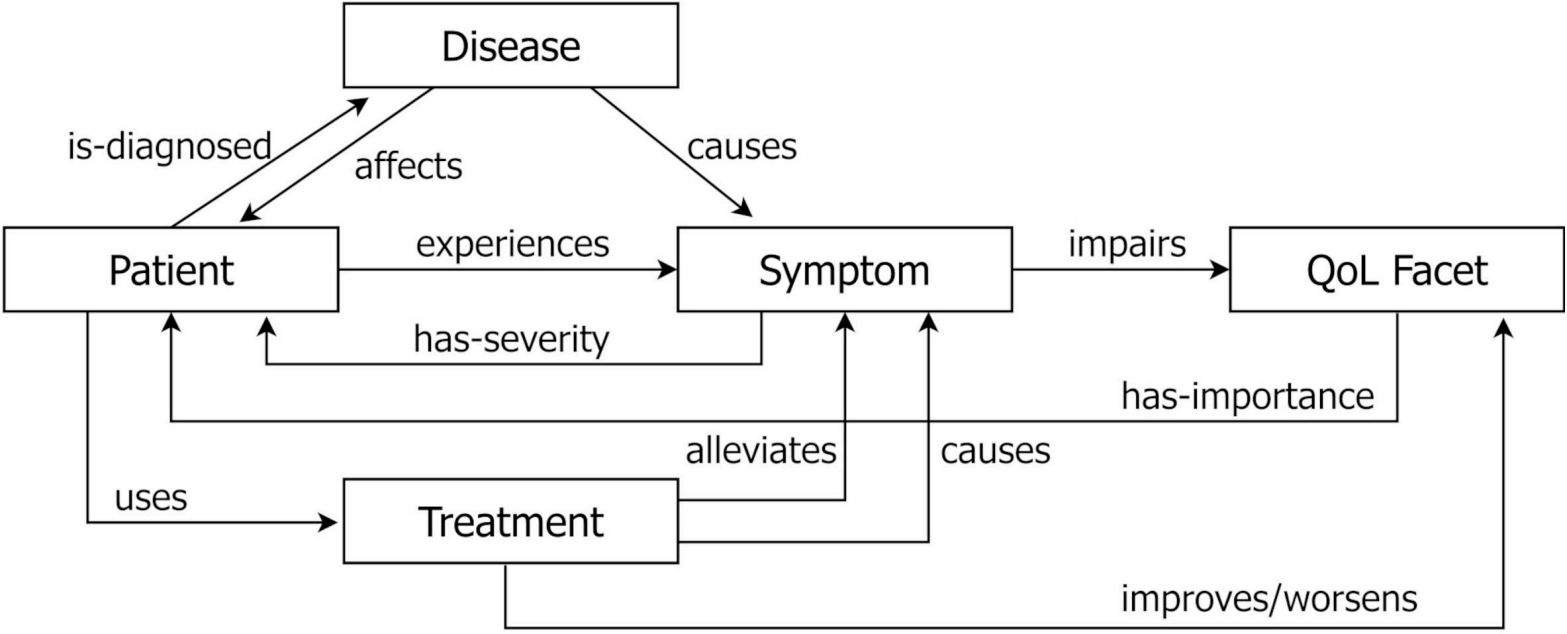
Schematic overview of the conceptual patient experience model (CPEM) to define the relevant information types/variables that can be extracted from patients’ posts in the context of SML studies. The five key elements (disease, patient, symptom, QoL facet, treatment) are linked to each other by conceptual relations as described in Table 3. The CPEM supports the choice of analysis method and NLP extraction algorithm used in a SML study.

Algorithmic coding of social media data often implies the use of NLP methods (67) in order to detect relevant entities such as symptoms (68), drug names (69), adverse effects (70, 71), among others. For many research questions, however, the most relevant aspects of the analysis are related to the impact of the burden of symptoms as well as the effectiveness and safety of treatments on QoL of patients. Thus, the most important insights can be gained from a relational analysis involving relations between different concepts such as symptoms, diseases, treatments on the one hand, and (QoL) improvements on the other. Depending on the research question, different relations will be relevant. To guide the choice of analysis and NLP extraction methods, we provide a Conceptual Patient Experience Model (CPEM) that represents some key concepts and the relations between these that patients typically report and discuss in social media. The model is depicted in Figure 2. This model can be seen as akin to the well-known PICO model that defines the key information (i.e., Patient, Intervention, Comparison, Outcome) that should be indexed for each clinical trial publication to support improved search (72). Along the same lines, our CPEM model attempts to define the relevant information types/variables that can be extracted from patients’ posts in the context of SML studies. In the case of CPEM, the consideration of all information items is not always mandatory as the selection of information items to extract will depend on the specific research project and question.

The CPEM consists of the following key elements:

- *Patient*: An identifiable (but pseudonymized) member of a cohort of patients under investigation, the experience of whom can be observed and collected based on their posts. Patient data can also be reported indirectly by others, such as caregivers, family, friends, etc.
- *Disease or condition*: The medical condition under investigation in the respective study that a patient suffers from.
- *Symptom*: Something that a person reports as a feeling or experience that may indicate that they are affected by a disease or condition.
- *Treatment*: A treatment is an intervention that health care providers or patients themselves apply to control a health problem, lessen its symptoms, or clear it up. This comprises drug and non-drug treatments, but potentially also lifestyle changes. Treatments can have different social and institutional status, such as being available only with prescription, over-the-counter, prescribed off-label, considered “alternative medicine,” illegal, etc.
- *QoL facet*: Individual aspects of patients’ experience in living with their condition in their real-world environment. QoL facets may be reported by patients as being impaired by a disease, symptoms or treatment. In case effective, a treatment may also be reported as improving aspects of QoL. Exemplary QoL facets concern the patients’ energy and fatigue, work capacity, mobility, personal relationships, sexual activity, among others (73). A related topic to QoL is that of the effect of the disease on activities of daily living (ADLs), which may affect a patient’s ability to maintain their lifestyle, such as finding a job or staying employed, studying, shopping, cleaning, etc.

The CPEM defines key conceptual relations between these concepts as described in Table 3, illustrated with valid textual examples that express the relation in addition to incorrect examples where this relation is not expressed.

**TABLE 3:** Key concepts of the conceptual patient experience model (CPEM).

Relation	Description	Valid example	Incorrect example
Affects	An individual patient in the cohort under investigation reports being affected by a certain condition. Here, a mere mention of the condition is not sufficient to substantiate an “affects” relation. Rather, an explicit contextual cue needs to be provided by a patient in their post in order to be able to infer an “affects” relation. Note that “affects” is of weaker veridicality compared to “is-diagnosed” (cf. below) in the sense that no explicit cue indicating a verified medical diagnosis is required in order to postulate an “affects” relation.	“I have last stage diabetic retinopathy and it’s one of the worst life changing diagnosis.”	“I turn 73 this year with all 10 toes and nearly perfect vision with no signs of retinopathy…”
Is-diagnosed	An individual patient in the cohort under investigation reports being diagnosed by a medical professional with the given condition or presenting proven diagnostic criteria (e.g., clinical laboratory test or readout, disease-specific prescribed medication). Cases of reported self-diagnosis are excluded from this definition, even if they may appear plausible in view of quoted signs or symptoms. Such cases may rather substantiate an “affects” relation (cf. above). Depending on the respective study objective and design, either an “affects” or an “is-diagnosed” relation may be more appropriate to serve as inclusion/exclusion criteria in selecting the patient cohort under investigation.	“So I went to a doctor and they told me that I have diabetes after testing my blood sugar in their office.”	“I have no diagnosis of diabetes, and I get a hbA1c done at least once a year as I’m registered with my GP”
Causes	This is a polyvalent relation linking either a Disease or Treatment to a symptom. It is not sufficient to have the Disease or Treatment mentioned in co-occurrence with the symptom (e.g., in the same post). Rather, an explicit contextual cue needs to be provided in order to be able to infer a “cause” relation.	“My left eye had more complications and swelling that caused my vision to be distorted and blurry for months—I actually had to have a steroid implant put in there.”	“Yes, I was able to keep my blood sugar well controlled for 7 years without insulin by eating a very low carb diet and a long honeymoon phase. I also have hypothyroidism.”
Experiences	This relation links an individual patient to a Symptom that the patient reports to have experienced, based on the presence of an explicit contextual cue that indicates the relation.	“And I have blurred vision this morning too!!”	“… Blurred vision is a common symptom around the time of a diabetes diagnosis and until blood glucose starts to stabilize.”
Has-severity	A patient may report a (often subjectively) perceived degree of severity in relation to an experienced symptom. Patient-reported severity levels can be captured in terms of directly reported values or normalized to discrete numerical or ordinal scales.	“I have this massive headache/migraines I’ve ever experienced in my life…”	“I tried it but it causes a headache, weakness and fever like feeling when I don’t eat carbs and am unable to do workout also.”
Uses	An individual patient reports using a particular treatment. A simple mention of the treatment by the patient is not sufficient. Rather, an explicit contextual cue needs to be provided in order to be able to infer a “uses” relation; this might include off-label uses of medications as well.	“I have been on Ozempic for about 2 years.”	“Ozempic is marketed for diabetes while Wegovy is marketed for weight loss.”
Impairs	A patient mentions that a particular symptom is impairing (i.e., negatively affecting) a particular aspect of their Quality of Life (QoL). Both concepts need to be contextually linked by an explicit linguistic cue indicating the “impairs” relation.	“I started getting random bouts of blurry vision, which escalated to the point I had to pull over and have my wife drive on the way back from vacation at night because I was functionally blind and couldn’t read a giant highway street sign 10 feet in front of our car.”	“I can relate to the blurry vision…”
Improves/worsens	A patient reports that a specific Treatment has improved or decreased a particular aspect of their QoL.	“The ozempic helped me with the initial weight loss for me to be able to move better.”	“My ozempic and glyburide did nothing to control my blood sugar…”
Alleviates	A patient reports that a certain treatment has been helpful in alleviating a symptom.	“I started on a daily iron supplement as recommended by my doctor which really helped with my feet not feeling like they were freezing constantly!”	“I have used saxenda in the past, and my first ozempic dose of 0.25 mg has had no effect that I’m aware of.”
Has-importance	A patient reports that a particular aspect of their QoL has a certain level of subjectively assessed importance to them.	“I have an amazing bf who took the time to understand how my blood sugars work, he’s not perfect at it but I see his effort and heart and that’s really what matters to me.”	“My girlfriend is a pharmacist so she’s able to work her magic with efficiently getting the cost down and dealing with insurance.”

From our experience, these are some of the most fundamental and most relevant relations when establishing a conceptual model of the disease following the *Concepts of Interest* [CoI; (74)] and *Meaningful Aspects of Health* [MHA; (75)] frameworks, although there might certainly be other specific relations that become relevant depending on the specific study objective. Therefore, it represents a core set that can form the foundation of any algorithmic analysis informing PFDD research questions. Additional relations that can be of interest but are not covered by the CPEM model can describe, for example, interactions between patients and other individuals (e.g., caregivers, HCPs, friends, family, co-workers, neighbors, classmates, strangers, etc.) as well as institutions and social constructs (e.g., hospitals, insurance companies, government agencies, employers).

### 4.2 Algorithmic coding via NLP algorithms

#### 4.2.1 Overarching principles

Large-scale analysis of online patient experience data requires algorithmic processing of patients’ online narratives using techniques from NLP, for which overarching principles can be defined as follows:

- Dedicated models must be in place to distinguish relevant stakeholder groups in social media data (e.g., patients vs. caregivers vs. HCPs).
- For relevant key concepts, as specified in the CPEM, it needs to be ensured that characteristics of the patients’ language are mapped to established sources of disease-specific medical nomenclature.
- Development of models for extraction of patient experience concepts should be guided by explicitly defined (annotation) guidelines, which should be derived from established research instruments to the greatest extent possible.
- Fundamental mechanisms should be in place for checking and monitoring the quality of extraction models. Acceptable performance thresholds should be specified prior to model training or development, respectively.
- As long as resulting data volumes are still acceptable, precision of extraction models should be given preference over recall, in order to avoid false positives.

#### 4.2.2 Main challenges

As a recurrent challenge, algorithmic approaches for SML need to be able to deal with variation in language use. This relates to at least three levels: (i) regular lexical and phrasal variation in language use, (ii) medical nomenclature vs. lay language, (iii) meaning alterations over time.

##### 4.2.2.1 Regular variation in language use

A relation as, e.g., “affects” between a disease and a patient can be expressed in many different ways, as shown by a small number of examples for diabetes below:

“I have been type 2 diabetic since 2014”

“When I was first diagnosed with diabetes it was over 400 mg/mL”

“How it’ll impact me as a T1D is the last of my worries honestly.”

##### 4.2.2.2 Medical nomenclature vs. lay language

Medical language is well defined and specified by medical nomenclature. However, the use of medical terminology in patient posts varies. E.g., symptoms are often described by sensation or everyday manifestation. Table 4 shows some examples of lay language descriptions of medical concepts.

**TABLE 4:** Examples for lay language descriptions of medical terms.

Medical term example	Lay language description
Diabetic neuropathy	“Hi—I had neuropathy for around 6 years. That included burning feet, particularly at night, stabbing “needle” pains, and pins and needles numbness.”
Nausea/gastrointestinal issues	“It was awful for me and I didn’t lose any weight. I would wake up to vomit at 4 a.m. It was horrible.”
Polyuria	“I had to drink a lot and pee a lot.”
Hypoglycemia	“Not one single doctor told me it could cause low blood sugar, it’s something I’ve had to figure out on my own!”

##### 4.2.2.3 Meaning alterations over time

As one of its key advantages, social media data bears the potential of capturing meaningful trends and alternations in online patients’ behaviors, preferences, perceptions or attitudes, as reflected in changing patterns of language use over time, e.g., because new medications were approved. In this case adaptation of the background of the NLP algorithms might be sufficient. In other cases where, e.g., references to medications alternate between the common drug name and the brand name, or creative neologisms are used to apply novel words to refer to the same things (e.g., “Vitamin O” for Ozempic), which can be considered a highly productive and unpredictable process of language variation, dynamic model adaptation methods might be neccessary.

While there is certainly no algorithm that can reliably recognize all possible variations discussed above, one needs to ensure that the selected algorithm has the capability to generalize, i.e., to recognize a reasonable set of variations. In the following subsection, we briefly review different classes of technical NLP approaches that are commonly used for algorithmic coding in SML, while also discussing their potential strengths and weaknesses in dealing with these specific challenges.

#### 4.2.3 Technical approaches

Various technical approaches can be used for developing NLP-based algorithms that can identify conceptual structures rooted in the CPEM (Section 4.1) and extracting them from online patient experience content. These can be roughly classified in several categories, although hybrid approaches are also possible:

- *Supervised machine learning models* follow the principle of learning meaningful patterns from large volumes of positive and negative training examples that are usually provided by human experts through manual annotation, based on detailed pre-specified annotation guidelines (76). In the present application context, supervised machine learning models usually have the form of relation extraction models that are trained to identify specific relations, such as those part of the CPEM, e.g., the “impairs” relation. Importantly, a separate extraction model needs to be trained for each relation type of interest. To obtain acceptable extraction performance, training of such models can be a costly iterative procedure. Supervised algorithms can be trained to reliably recognize variability, but the capacity to do so crucially depends on the variation they have seen in the training data. When assessing the suitability of a supervised algorithm, ensure to gain some understanding of the processes and phenomena governing linguistic variability that is observable in the training data.
- *Rule-based models* can be applied to similar problems as supervised machine learning models, with the main difference that they need to be fully manually specified in terms of explicit rules (similar to “if/then” statements in programming languages). On the one hand, therefore, rule-based models exhibit the highest possible degree of transparency and precision. On the other hand, their main challenge is to cope with high degrees of linguistic variability, which needs to be directly encoded into a multitude of rules (e.g., to enable the model to identify all possible variants of expressing an impairment relation). Hence, rule-based extraction models are generally known to suffer from severe coverage issues. They rely on explicit human expert encoded lexicalizations of concepts that are pre-defined in the rules and can only deal with variability, if it has been explicitly encoded and applied to sentiment analysis (77).
- *Language models (LMs)* can be applied as extraction models for patient experience content by capitalizing on an appropriate foundational model and adapting it to the (parts of) conceptual structures of interest via fine-tuning or prompting (78). This category includes transformer-based language models such as BERT as well as large pre-trained models such as ChatGPT with billions of parameters, so called Large Language Models (LLMs). While standard task-specific supervised models generally have a higher performance than LLMs on individual biomedical NLP tasks relevant for patient experience research (79, 80), LLMs have the advantage of being extremely flexible with respect to the specific extraction task at hand. As the only requirement in this setting, a meaningful prompt statement needs to be specified by a domain expert to define the extraction task for the LLM. As a particular strength, LLMs are highly versatile in dealing with linguistic variation, since they have been pre-trained on extremely large amounts of text and have, therefore, acquired substantial “linguistic knowledge.” As a drawback, compared to the above-mentioned models, there is typically no way to inspect or “debug” the model to understand which lexical variations it is recognizing and which not. In fact, large-scale validation of LLM results is still an open issue which may incur considerable additional costs in complex scenarios or when large volumes of LLM output need to be validated. A survey of work applying LLMs to information extraction has been recently provided by Xu et al. (81).
- *Unsupervised approaches* such as data clustering (82) or different variants of latent semantic analysis (83) or topic modeling (84) can be applied to find trends or patterns in the data that do not correspond to pre-defined concepts. While unsupervised methods cannot recognize specific concepts or relations as defined in the CEPM, they can be used to detect higher-level trends.

Table 5 summarizes the most common NLP-based methods and their applicability and limitations.

**TABLE 5:** NLP-based methods, advantages, and limitations.

Methods	Best for	Limitations
Rule-based models	High precision, small datasets	Poor scalability, brittle rules, risk of missing out information
Supervised Learning	Large datasets, specific tasks	Needs annotated training data
(Pre-trained, Large) language models	Versatility, rapid deployment	Cost, validation challenges, tuning the precision/recall tradeoff
Unsupervised approaches	Discovering trends and patterns in data	Not suited to extract specific facts/relations as identified in the CPEM

#### 4.2.4 Validation methods

With respect to rule-based and supervised models, it is an established best practice to evaluate model outputs against ground truth data, usually manually annotated by humans, in terms of precision and recall or accuracy figures. For example, a supervised machine learning model designed for the task of classifying disease-related content into documents that are authored by patients or other stakeholders (e.g., caregivers, medical professionals) would require a relevant volume of sample documents to be annotated according to their membership with either class for the model to learn criteria for automatic classification of documents into the “patient-authored” vs. “other” scheme.

The major proportion of an annotated data set is typically used for training or building an initial model, while smaller subsets of the data are set aside for model optimization and evaluation. This separation ensures that a relevant fraction of data points has not been seen by the model during development or optimization phases, thus allowing to assess the robustness and generalization capabilities of the model. Common proportions of data splits are 80% of the data being used for training, while 20% are reserved as development or evaluation sets (67).

The same approach can be used for LLMs when they are applied to “closed” tasks with a fixed output schema (as in the patient classification example above), even though the variability in their output generation provides some level of difficulty. In more flexible summarization (78) or question-answering (QA) scenarios, though, pre-compiled gold standard data sets are obviously not sufficient to cover the potential “open-endedness” of queries/prompts to the LLM. Validating LLMs in these scenarios is an open research question. Preliminary methods and benchmarks currently available are Ribeiro et al. (85), Wu et al. (86), Big Bench (87), or HELM (88). These approaches have in common that they test the capabilities of a productive LLM to deal with general linguistic phenomena in natural language data, while abstracting from concrete application domains or problems. To address the latter dimensions, these validation approaches would need to be extended to the respective domain at hand.

### 4.3 Descriptive statistics and quantitative analysis

**FIGURE 3:**
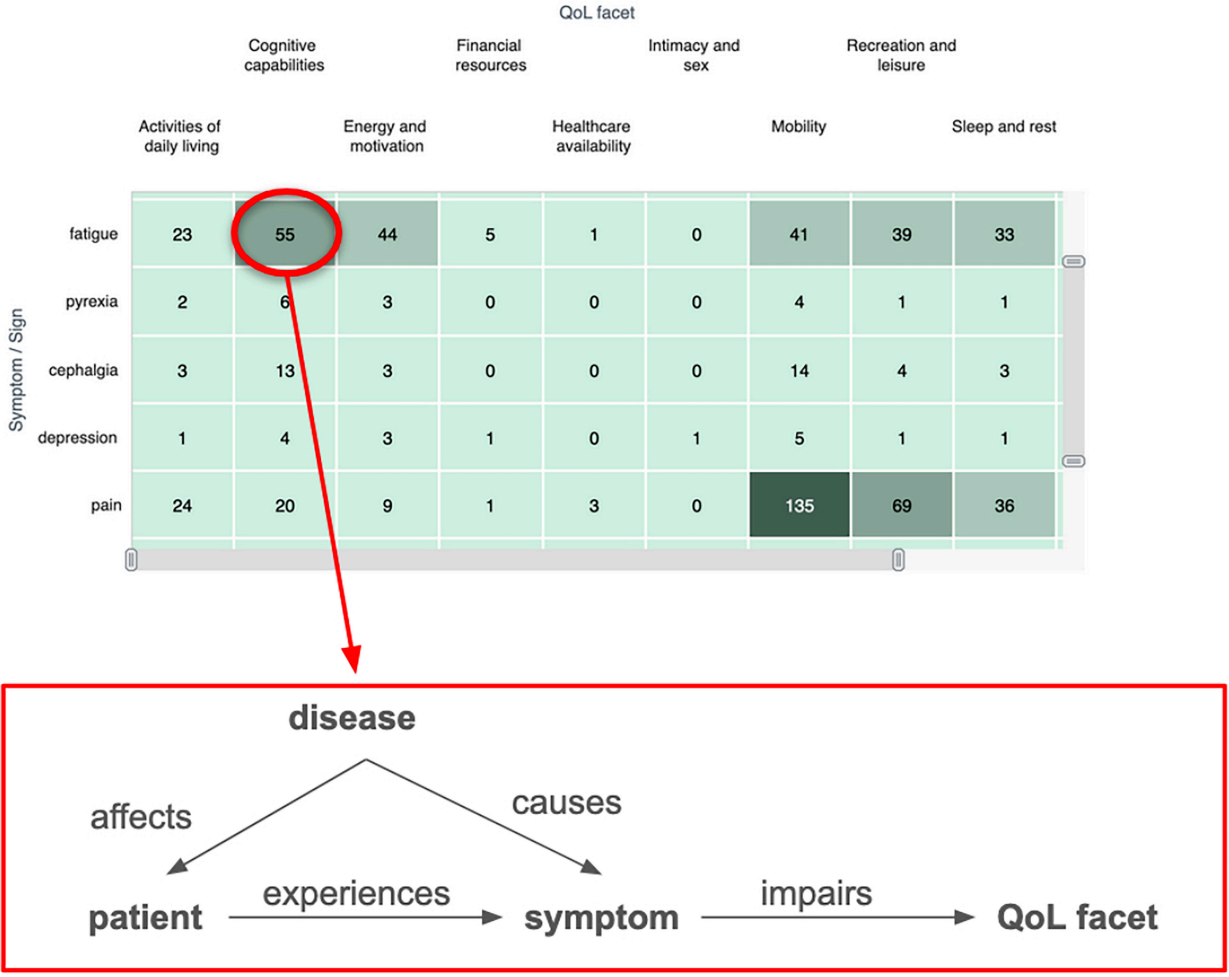
Example of a heat map display of descriptive statistics from an unpublished SML study. Symptoms as mentioned by patients in social media posts are captions of rows on the left side of the table, while QoL facets are captions of the columns. The numbers indicate pairwise mentions of a certain symptom with a QoL facet, for which a dedicated algorithm inferred a patient-reported cause-effect relation. Dark shading of a cell indicates a high number of detections. In this example, “pain” often impaired the QoL facet “mobility.” As depicted in the CPEM overview below, this table shows a descriptive statistical representation of the relation between “symptom” and “QoL facet.”

Based on the relations extracted by large-scale algorithmic analysis, we can create descriptive statistics, showing the frequency with which certain relations are reported, e.g., the number of patients that report a particular set of symptoms or a treatment, the distribution of symptom severity levels as experienced by patients, how frequently patients mention that a treatment alleviated a particular symptom, etc. Figure 3 depicts an example for a descriptive statistical output of these data.

Beyond these more descriptive statistics, inferential statistics can be computed based on statistical (hypothesis) tests. Such tests would typically assume a set of hypotheses (null hypothesis vs. alternative hypothesis) and rely on an appropriate statistical test to potentially reject the null hypothesis in favor of the alternative hypothesis. Multiple parametric (e.g., *t*-test) and non-parametric tests can be applied here (e.g., Kruskal Wallis). In addition to such tests, (linear) regression or factor analysis can also be applied to uncover statistical associations in the data. As an example, one might analyze whether there are statistically significant differences between the symptoms experienced by two cohorts or sub-cohorts (e.g., of different age, gender, comorbidities, treatments, etc.) using a hypothesis test [for further information on statistical analysis of categorical data see Bilder et al. (89)]. In addition, regression analysis might be applied to discover statistical associations between certain symptoms and QoL aspects (90). In a recent publication, it has been shown how correlation analysis can be applied to structured variables extracted by SML.^9^ The study quantified how the mention of cognitive symptoms is correlated with the mention of specific QoL facets in Schizophrenia. Data extracted from social media does, therefore, not differ from any other (structured) variable obtained via interviews, questionnaires etc. in terms of statistical analysis.

### 4.4 Qualitative analysis

Qualitative research aims at understanding complex social phenomena in depth or to gain insight into people’s experiences and perspectives, often by investigating the meaning people ascribe to their experiences or underlying reasons for their behavior or preferences. In the context of patient experience research, qualitative data analysis can be used to identify themes in patient language, potential connections between themes or emerging themes. Such themes may comprise all facets of the CPEM described above, i.e., experiences that patients make along their journey of living with a disease, existing patterns of treatment use, how certain symptoms are subjectively experienced or affect their QoL, including activities of daily living. Qualitative analysis can also reveal insightful patterns of the language patients use to describe these concepts and experiences. While qualitative analysis has traditionally relied on manual workflows, recently it has been shown that LLMs have strong potential to automate certain steps in qualitative research workflows (91, 92), and can be used to reliably summarize the patient conversation on a particular topic (78). Thus, qualitative data, such as description of feelings and subjective experiences can be, to some extent, mapped with approaches such as sentiment analysis and the use of qualitative scales.

### 4.5 Recommended protocol of procedural steps in analyzing online patient experience data

We propose a common workflow of procedural steps for NLP-driven analysis of online patient experience data in the following. As a starting point, we assume a comprehensive data corpus (i.e., a collection of thematically relevant, usually disease-specific, social media posts) being available as a result from a previous data collection step (cf. Section 3).

1. Determine author type for each post; select only patient-authored (or indirectly provided by caregiver, family, etc. as pre-specified) content from the disease-specific data corpus.
2. Determine appropriate diagnostic criteria to be fulfilled by the patient. Aggregate relevant patient posts into pseudonymized patient records.
3. If available, determine demographic variables, i.e., author’s age at the time of reporting or diagnosis, author’s gender, and other relevant filter variables.
4. Select records according to demographic variables or inclusion/exclusion criteria of interest to create a cohort of “algorithmically identified” patients.
5. Select relevant concepts and relations from the CPEM and corresponding algorithms that can extract them with the desired level of accuracy, robustness, coverage etc.
6. Triangulate findings from social media with results from other studies involving clinical data or other sources of RWD to confirm hypotheses.
7. Present results in terms of appropriate statistics, e.g., descriptive statistics in terms of ranking, frequency distribution of top-n most frequently reported conceptual structures, or inferential statistics in terms of significance testing between cohorts investigated.
8. Alternatively, or in addition, apply manual or algorithmic workflows for more in-depth qualitative analysis on top of the results produced in previous steps.

This new perspective is discussed within the Pistoia Alliance community and poses an option that could be explored in alignment with practices from the field of qualitative studies.

When running the above process, ensure to establish procedures for reporting adverse events, off-label uses etc. following your own organizational process and requirement for pharmacovigilance (PV) reporting. PV reporting obligations do not differ for social media listening compared to other types of patient research and follow the guidelines by the European Medicines Agency (EMA). The employees of organizations obliged to report adverse events, pharmaceutical manufacturers in particular, are obliged to do so within one business day of getting to know the adverse event. In the context of social media listening, reporting duties are limited to cases where someone actively and consciously reads a social media post that contains such an adverse event. The minimal criteria to be satisfied to be reportable are typically defined in organization-specific standard operative procedures for PV reporting that might differ across organizations. There is no reporting obligation for AEs that are discovered via algorithmic processing but have not been inspected by a human analyst.

### 4.6 T2DM example use case: mixed methods for analyzing the data

In our case study, we applied four different algorithms:

1. A supervised algorithm to classify whether a post comes from a patient, caregiver, an HCP or none of these.
2. A rule-based algorithm identifying statements of patients self-reporting to suffer from or have been diagnosed with T2DM with appropriate synonyms.
3. A pattern-based algorithm detecting statements in which patients report their HbA1c values.
4. A supervised relation classification algorithm that detects the “severity” with which a certain comorbidity impacts patients’ lives, mapping each symptom or comorbidity report into a discrete severity scale consisting of low, middle and high severity.

*The above algorithms are applied to the set of algorithmically identified patients that satisfy the following inclusion criteria:*

1. Posts that were published during the observation period of 28.02.2023–28.02.2025;
2. Patients explicitly mention suffering or having been diagnosed with T2DM in at least one post, or;
3. They mention that they have a HbA1c ≥ 6.5% ( ≥ 48 mmol/mol) (93).

*Patients or documents were excluded if:*

**FIGURE 4:**
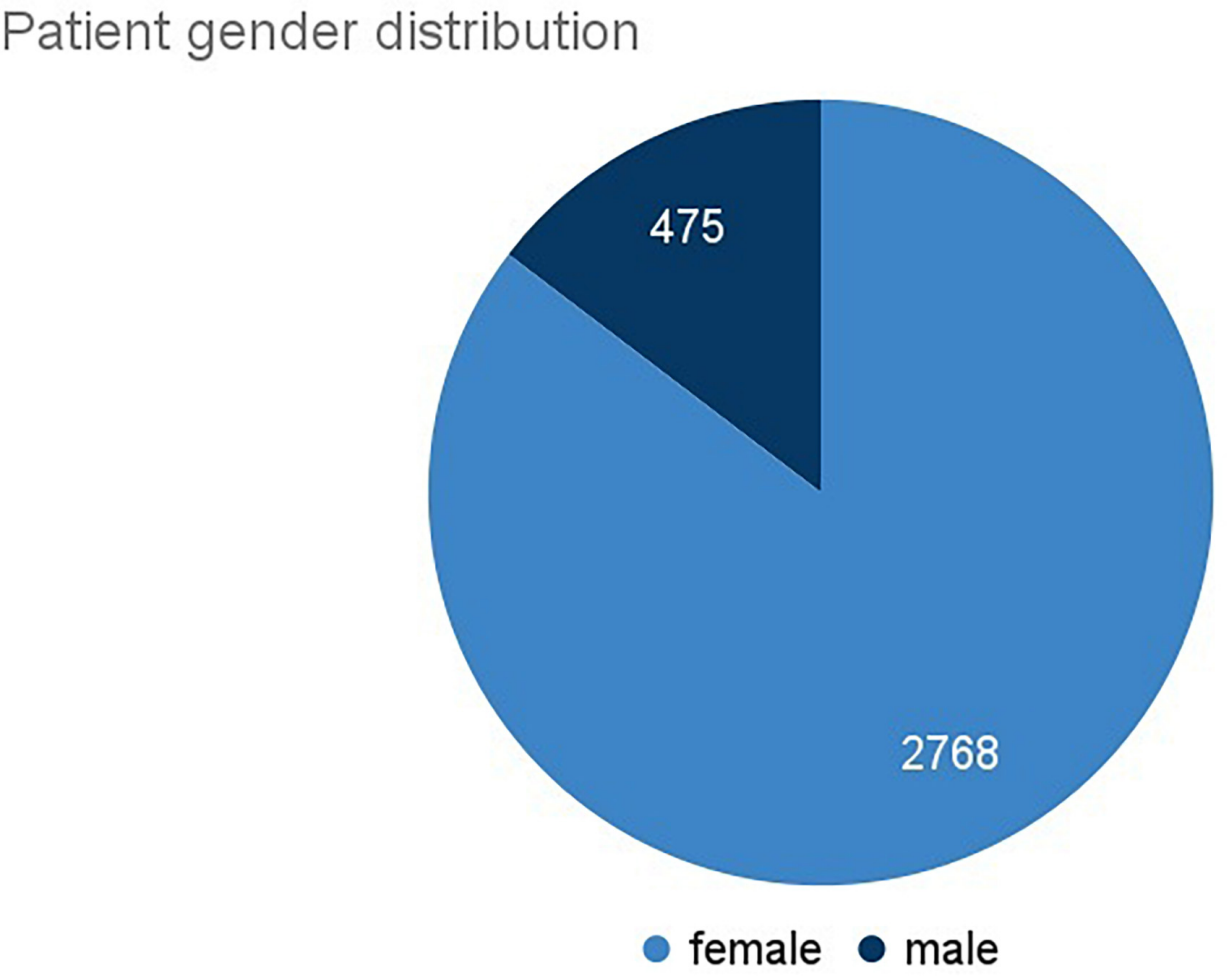
Pie chart of the author-reported gender distribution in the T2DM example case study.

1. The patient authored more than 5,000 posts within the 2-year observation period. This threshold was set purposively after manual review of authors with extraordinary high post volume revealed these as automated advertisements;
2. A document was authored outside the 2-year timeframe 28.02.2023–28.02.2025;
3. The patient received an A1C label “Unidentified” or “Normal A1C” indicating that it is not a diabetes patient;

As a result, we obtained 527,268 English language datapoints from 11,678 different authors. The gender distribution of this cohort is shown in Figure 4, showing that there is a bias toward a female population. We collected gender information for patients that explicitly report it, e.g.

*“I am a male T2DM patient with age 36,”*

*“I’m 48 year old female, type 1—so three of the risk factors,”*

*“I’m a 64 YO male T2. I was diagnosed about 25 years ago.”*

*Data analysis:* In this case study, we used solely machine-learning models and not human annotations and applied a mix of quantitative and qualitative analysis approaches. To answer the first research question of this use case, we rely on the model identifying the subjectively perceived severity for each comorbidity as defined below.

- *High severity:* Indicated by linguistic cues emphasizing symptoms, such as intensifying adverbs, comparisons, repetitions, or capitalization, and descriptions highlighting effects on daily activities. Example: *“I am close to stage 4 diabetic retinopathy, and I am almost unable to see where I am going…”*
- *Low severity:* Characterized by language that downplays the disease’s effects or provides cues suggesting insignificance. Example: *“I was told that I have mild diabetic retinopathy which had not bothered me at all…”*
- Medium severity: Applied when a disease is mentioned without specific emphasis on its effects or clear cues for either high or low severity.

**FIGURE 5:**
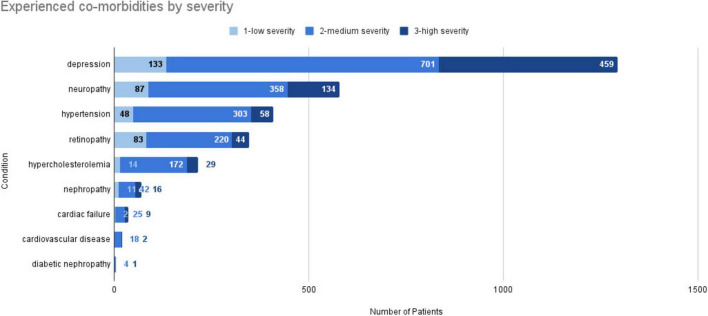
Bar chart of the experienced comorbidities by severity in the T2DM example case study. This figure shows the total number of patients reporting a certain symptom, broken down into three severity levels: 1, low; 2, medium; and 3, high. The severity was assessed automatically as described in the text.

The categorization into severity levels allows quantification of posts stating this level of patient experience as shown as bar chart in Figure 5.

It is evident that depression is by far the most frequently reported comorbidity with a relatively large share of “high severity” statements. Other comorbidities such as neuropathy, hypertension, retinopathy, and hypercholesterolemia are less frequently reported and present with a lower share of high-severity. Overall, such findings allow us to get a clearer picture of the disease burden that patients face, and which comorbidities are most impactful. These insights can thus support the creation of a conceptual model of the disease and help to focus drug development and care on those symptoms and comorbidities that are most burdensome.

**FIGURE 6:**
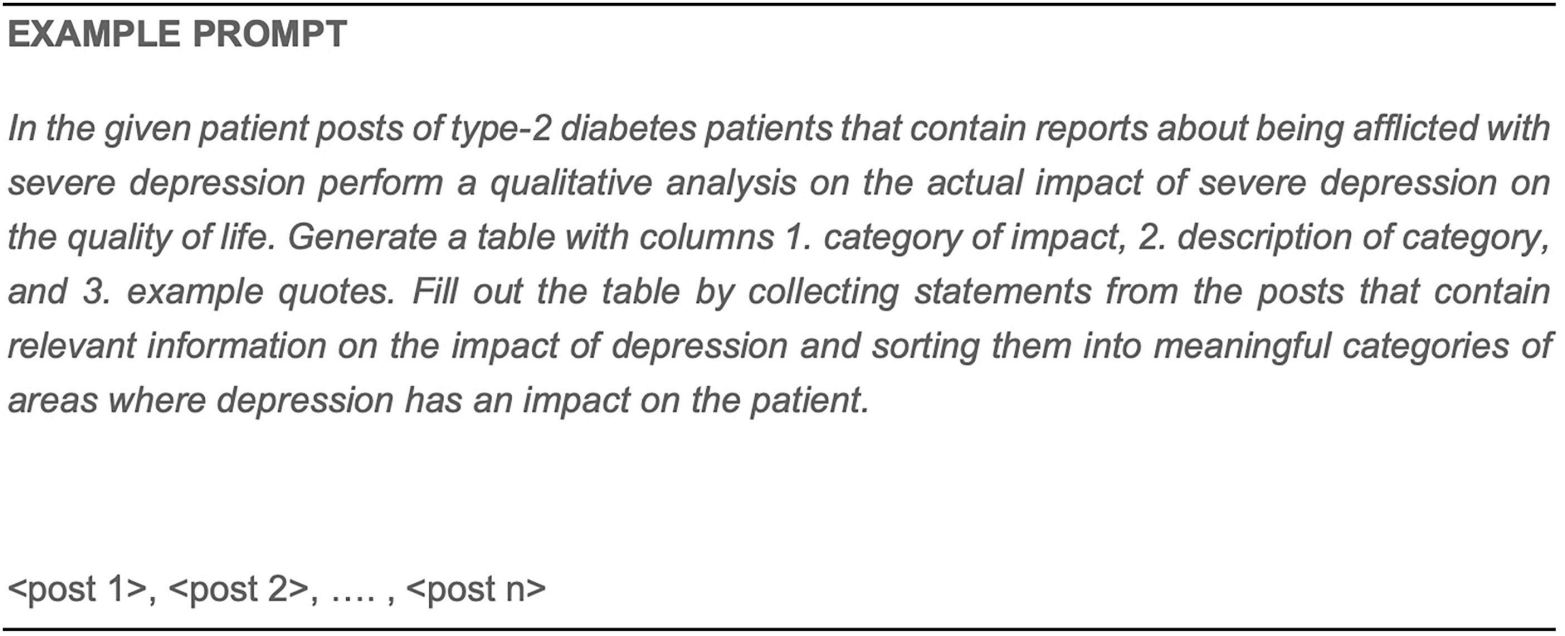
Example prompt for summarizing online patient experience reports in a tabular format.

Regarding the second research question, we use an LLM to examine the impact of each symptom/comorbidity separately. As an example, we show how we can obtain a qualitative summary of the impact of depression on the daily activities of patients by using a retrieval-augmented generation (RAG) approach (94) and having an LLM automatically summarize the relevant documents describing the impact of depression, following the methodology proposed by Nair et al. (78). In this example study, the prompt shown in Figure 6 was used. Table 6 shows the automatically generated summary for the impact of depression on the daily life of T2DM patients. It shows example quotes from patients that have been paraphrased to preserve anonymity.

**TABLE 6:** LLM generated summary highlighting various areas of impact and example quotes of collected PED.

Area of impact	Description	Paraphrased example quotes
Emotional wellbeing	The overall emotional state and feelings of sadness or hopelessness.	“I constantly feel a sense of sadness. I deeply long for my previous life and the freedom I once experienced.”
Daily functioning	The ability to perform daily tasks and responsibilities.	“I struggled to complete that task. I would spend hours in anguish just thinking about finishing it.”
Motivation	The drive to engage in self-care activities, including diabetes management.	“When I’m experiencing a depressive episode, it certainly complicates things… I lose interest in activities I once enjoyed.”
Social interaction	Relationships with others and social engagement.	“I occasionally have fleeting moments of feeling okay… but overall, I don’t feel joy.”
Physical health	The impact on physical health due to neglect or poor management.	“My blood sugar levels fluctuate between 10 mmol and 16/17 mmol, which I understand is too high… My body struggles to combat infections, and I constantly feel unwell.”
Self-esteem	Feelings of worthlessness or inadequacy related to diabetes management.	“I feel so shattered; is she truly investing her time in someone like me?”
Coping mechanisms	Strategies used for dealing with depression, such as food choices.	“Managing my diabetes has made comfort eating a challenge; some days I barely eat, while on others, I overindulge.”

Taken together, this case study shows how we can apply the best practices described in this article to perform a quantitative and qualitative SML study to learn about the perceived severity of key symptoms and comorbidities of T2DM patients and how they affect their daily lives.

**Best practices/recommendations:**

- Use the CPEM framework to select concepts and relations relevant to the research objective/question. If needed, extend the model to capture specific aspects/concepts/relations that go beyond the CPEM in its currently proposed generic version.
- Ensure to extract specific/explicit relations rather than only mentions of concepts to increase precision/accuracy.
- Select a suitable set of algorithms to extract the relevant concepts/relations on the basis of the required precision/accuracy /coverage etc.
- Define background characteristics (demography, diagnosis, etc.) that can be algorithmically evaluated to include patients into the study.
- Ensure that selected models and algorithms can deal with lexical variation adequately.
- Validate the selected algorithm by measuring precision/recall on a sample of test data.
- Follow the proposed 7-step analysis workflow, modify it as required by the research question/objective.
- Combine different methods and data to confirm findings.
- Ensure to establish procedures for reporting adverse events, off-label uses etc. following your own organizational process and requirements for PV reporting.

## 5 Conclusion

Social media plays an important role for patients as they receive emotional, network and information support that helps them in coping with their condition. They share rich experiences in public conversations with their peers that can help us to better understand how their disease affects their daily life, which unmet needs they have, what outcomes make a difference to them, and what experiences they make with treatments. Thus, regulators worldwide have explicitly highlighted that social media can be an important source of patient experience data and inform patient-focused drug development activities. Yet, there are no generally agreed-upon best practices and methodologies for how to leverage social media data for patient-focused drug development activities that can meet regulatory requirements. This article has attempted to reduce this gap by clearly outlining what advantages social media listening (SML) has compared to other research methods, while at the same time highlighting limitations. We have also outlined best practices in terms of choice of research settings and objectives that SML studies support, and we have proposed best practices for the collection and analysis of social media data. We have illustrated the application of these best practices using a small example case study with the goal of understanding the impact of comorbidities on T2DM patients. One limitation of the study is that results have neither been triangulated with other methods nor validated in discussions with patient organizations or representatives. In ongoing work, the Pistoia Alliance group on “Social Media Listening for PFDD” is engaging with patients to understand their awareness of and perspectives about SML being applied to online public data.

With this work we thus contribute to the clarification of the role that social media can play in patient-focused drug development and provide concrete guidelines for those wanting to adopt SML as part of their evidence generation activities. As future perspective, the presented guidelines which were developed by experienced users of SML in PFDD, should be discussed with a broader relevant scientific society and also with regulators (e.g., EMA, MHRA, Medicines and Healthcare products Regulatory Agency), in order to provide valuable standards to the field of social media research.

Overall, this opens an avenue for using SML to inform patient-focused drug development that can meet the needs of regulatory decision making and ultimately contribute to focus on outcomes that truly matter to patients and have the potential to significantly improve their lives.

## Footnotes

1
https://www.fda.gov/drugs/development-approval-process-drugs/fda-patient-focused-drug-development-guidance-series-enhancing-incorporation-patients-voice-medical

2 Concept of interest as defined by the FDA (https://www.fda.gov/drugs/development-approval-process-drugs/patient-focused-drug-development-glossary#:~:text=Concept%20(also%20referred%20to%20as,Endpoints%20and%20Other%20Tools)%20Resource%20)

3
https://www.pistoiaalliance.org/news/pistoia-alliance-launches-new-community-of-experts-to-use-social-media-insights-to-drive-patient-centric-drug-development/

4
https://gdpr.eu/

5
https://oag.ca.gov/privacy/ccpa

6
https://en.wikipedia.org/wiki/Personal_Information_Protection_Law_of_the_People%27s_Republic_of_China

7
https://www.legislation.govt.nz/act/public/2020/0031/latest/LMS23223.html

8
https://aoir.org/reports/ethics3.pdf

9 Poster presentation: Wieckowski et al. (90).
