# Supplementary material for: Best practices for the collection and analysis of patient experience data from social media for patient-focused drug development
Source: Front Med (Lausanne). 2026 Jan 30;12:1703923. doi: 10.3389/fmed.2025.1703923 (PMC12900760; doi:10.3389/fmed.2025.1703923)
Supplement: Supplementary file 1 [file Table_1.docx]

Supplementary Table 1: Online Forums included in the example case study.

| **Forum** | **Number of posts** | **Parent Forum / Site** | **Comment on Terms of Service** |
| --- | --- | --- | --- |
| General Message Board | 27,095 | forum.diabetes.org.uk | Terms of Service of [diabetes.org.uk](http://diabetes.org.uk) allow for crawling data without an account |
| Newbies say hello here! | 11,288 |  |  |
| Food/carb queries + recipes | 3,601 |  |  |
| Pumping and Technology | 1,751 |  |  |
| In the news... | 1,717 |  |  |
| Entertainment - jokes, quizzes, funny pictures | 1,419 |  |  |
| Community - Chat with People about Remission | 1,053 |  |  |
| Diabetes & More | 787 |  |  |
| The Weight Loss Group | 681 |  |  |
| Exercise/Sport | 451 |  |  |
| Happy Birthday | 370 |  |  |
| Diets - Food Ideas and Discussions | 187 |  |  |
| Parents | 153 |  |  |
| Driving/DVLA | 132 |  |  |
| Recommended books | 98 |  |  |
| Pregnancy | 84 |  |  |
| Weight Loss Journey | 81 |  |  |
| Weight Loss Jabs | 76 |  |  |
| Stigma | 59 |  |  |
| Recipes | 30 |  |  |
| Diabetes in School | 29 |  |  |
| Events | 25 |  |  |
| Links | 19 |  |  |
| Heroes and Heroines! | 3 |  |  |
| Screening for type 1 community chat | 1 |  |  |
| Bariatric Surgery Discussion | 1 |  |  |
| diabetes | 125,624 | reddit.com | Terms of Service of [reddit.com](http://reddit.com) state that posts are public data and can be crawled via licensed providers |
| diabetes_t1 | 123,437 |  |  |
| Ozempic | 77,005 |  |  |
| Type1Diabetes | 72,642 |  |  |
| diabetes_t2 | 66,671 |  |  |
| type2diabetes | 10,698 |  |  |
